# The key role of depression and supramarginal gyrus in frailty: a cross-sectional study

**DOI:** 10.3389/fnagi.2023.1292417

**Published:** 2023-11-09

**Authors:** Sara Isernia, Valeria Blasi, Gisella Baglio, Monia Cabinio, Pietro Cecconi, Federica Rossetto, Marta Cazzoli, Francesco Blasi, Chiara Bruckmann, Fabrizio Giunco, Sandro Sorbi, Mario Clerici, Francesca Baglio

**Affiliations:** ^1^IRCCS Fondazione Don Carlo Gnocchi ONLUS, Milan, Italy; ^2^Fondazione Istituto FIRC di Oncologia Molecolare, Milan, Italy; ^3^Department of Pathophysiology and Transplantation, University of Milan, Milan, Italy

**Keywords:** frailty, aging, depression, cognitive impairment, brain, MRI, supramarginal gyrus

## Abstract

**Background:**

The age-related decrease in reserve and resistance to stressors is recognized as frailty, one of the most significant challenges identified in recent years. Despite a well-acknowledged association of frailty with cognitive impairment, depression, and gray matter morphology, no clear data are available regarding the nature of this relationship. This cross-sectional study aims to disentangle the role of the behavioral, neuropsychological, and neural components as predictors or moderators of frailty.

**Methods:**

Ninety-six older adults (mean age = 75.49 ± 6.62) were consecutively enrolled and underwent a clinical and MRI (3 T) evaluation to assess frailty, physical activity, global cognitive level, depression, wellbeing, autonomy in daily living, cortical thickness, and subcortical volumes.

**Results:**

Results showed a full mediation of depression on the link between cortical thickness and frailty, while the cognitive level showed no significant mediating role. In particular, left supramarginal thickness had a predicting role on depression, that in turn impacted frailty occurrence. Finally, handgrip weakness was an early key indicator of frailty in this study’s cohort.

**Conclusion:**

These data substantiate the role of depression in mediating the link between neural integrity of the supramarginal gyrus and frailty. In the complexity of frailty, handgrip weakness seems to be an early key indicator. These results are relevant for the design of rehabilitation interventions aimed at reversing the frail condition.

## 1. Introduction

The age-related decrease in reserve and resistance to stressors is recognized as frailty, one of the most significant global public health challenges in recent years due to the increase in the life expectancy in the general population (Dent et al., 2019). Frailty in older adults leads to significant vulnerability to adverse events and reduced ability to recover from health issues (Clegg et al., 2013).

Frailty has been conceptualized according to two principal models: the accumulative deficits multidimensional model, initially conceptualized by Mitnitski et al. (2001), and the frailty phenotype, proposed by Fried et al. (2001). The first is based on the Frailty Index (Rockwood and Mitnitski, 2007), calculated based on the degree of accumulation of health deficits, including comorbidities, psychological factors, symptoms, and disabilities. The model of the frailty phenotype, instead, defines frailty as an independent syndrome based on five physical signs/symptoms: poor handgrip strength, slow gait speed, involuntary weight loss, exhaustion, and sedentary behavior (Fried et al., 2001). The frailty phenotype has been reported as a potential transition state between healthy and pathological aging, plausibly anticipating disability (Bandeen-Roche et al., 2006; Cheung et al., 2018). In the present study, we focused on this latter conceptualization of frailty as a medical syndrome (Bandeen-Roche et al., 2006; Xue, 2011; Dent et al., 2019) whose mechanisms involved are yet to be defined. This approach was considered more suitable for identifying risk factors in this transition state with consequent relevant implications for timely and effective therapeutic strategies.

Despite a well-acknowledged association of frailty with cognitive impairment (Kelaiditi et al., 2013; Ruan et al., 2015) and depression (Soysal et al., 2017), no clear data are available regarding the nature of this relationship. Specifically, several contributions investigated a potentially reversible condition, cognitive frailty, that is the simultaneous presence of both physical frailty and cognitive impairment (Kelaiditi et al., 2013; Ruan et al., 2015), leading to an enhanced risk of neurocognitive disorders (Avila-Funes et al., 2012; Panza et al., 2015a,b), functional disability, poor quality of life, and mortality (Sternberg et al., 2011; Feng et al., 2017; Sugimoto et al., 2018). Moreover, several studies (Avila-Funes et al., 2009; Bunce et al., 2019) advocated similar risk factors for physical frailty and cognitive impairment, such as lack of physical activity, reduced social stimulation, and higher hospitalization. Furthermore, several studies highlighted the link between depression and frailty (see Soysal et al., 2017 for a review and meta-analysis), but the direction of this relation is still under debate. Depression has been considered somehow a consequence of frailty or an overlapping syndrome due to the phenotypic similarity sharing the loss of energy, fatigue, poor sleep, and reduced interest (Mezuk et al., 2012; Buigues et al., 2015; Canevelli et al., 2015; Bunt et al., 2017). Additionally, other evidence considered depression and frailty as interrelated, with each condition representing a risk factor for the other (Chang et al., 2010; Soysal et al., 2017).

In this framework, the investigation of neural integrity can help in disentangling the causal relationship between frailty phenotype, cognitive level, and depression.

From a neurobiological perspective, frailty syndrome appears to be accompanied by changes in the microstructural integrity of cortical and subcortical gray matter (Avila-Funes et al., 2017; López-Sanz et al., 2018; Maltais et al., 2020; Tian et al., 2020). Moreover, in frail subjects, reduced brain volume (Del Brutto et al., 2017) has been shown in regions important for cognition and emotion processing, such as the hippocampus, amygdala, fusiform gyrus, medial prefrontal, and orbitofrontal cortex, inferior frontal gyrus, primary somatosensory cortex, insula, superior temporal sulcus, and cerebellum. Furthermore, the mean cortical thickness of areas involved in mobility and neurodegenerative diseases has also been linked to frailty (Lu et al., 2020). Finally, several studies showed a strong association between frailty and cerebrovascular disease explored in terms of white matter hyperintensities (WMH) (Avila-Funes et al., 2017; Siejka et al., 2020; Ducca et al., 2023).

To summarize, the frailty phenotype is strictly related to cognitive decay, depression, and loss of neural integrity. What remains to be clarified is the type of relationship between these factors. In this line, our study aimed to identify the best predictors of the frailty phenotype among cognitive impairment, depression, and loss of neural integrity and to examine their causal relationship. To achieve this aim, we characterized a cohort of 96 community-dwelling older adults.

## 2. Methods

A cross-sectional study was performed.

### 2.1. Participants

Participants were recruited considering the following inclusion criteria: (i) age > 65 y; (ii) Mini-Mental State Examination (MMSE) score > 18 [according to Pezzotti et al. (2008)] to exclude severe dementia; (iii) absence of MRI exam contraindications (i.e., pace-maker, or other not-MRI compatible metallic implants or prosthesis); (iv) signed the informed consent module approved by Don Gnocchi Foundation Ethics Committee; (v) absence of diagnosis of Parkinson’s Disease, Alzheimer’s disease or infectious disease; (vi) absence of an unstable condition of a cardiac, vascular, pulmonary, hepatic, renal, endocrine, hematological disease; (vii) no drug and/or alcohol abuse; (viii) absence of an unstable psychiatric condition.

They were consecutively enrolled between 2019 and 2022 at the Don Gnocchi Foundation Institute, both at the Palazzolo Institute and the IRCCS *S. Maria* Nascente Rehabilitation and Care clinic: they were community-dwelling older adults attending the rehabilitation and care service at the center, available to participate in the study after physician proposing the enrolment in the research or after reading information flyers at the clinic or after word of mouth. Participants could be either attending the clinic for any health-related problem such as memory complaints, cardiac or pulmonary disease, or orthopedic problems or could be volunteers operating at the clinic or informal caregivers accompanying a patient.

### 2.2. Procedure

The study’s participants were involved in the research by taking part in a single session lasting about 1.5 h. The session included (1) a clinical and neuropsychological evaluation by a physician and a neuropsychologist and (2) a brain structural MRI examination.

#### 2.2.1. Clinical and neuropsychological evaluation

For the clinical evaluation, each participant was screened in terms of the frailty phenotype (Fried et al., 2001) according to Fried’s criteria (unintentional weight loss ≥4.5 kg in the prior year; grip strength measured with a manual dynamometer in the lowest 20% according to gender and Body Mass Index (BMI); poor endurance/exhaustion; walking time in the slowest 20% adjusting for gender and height using the 10 M walking test (Bohannon, 1997); kcal/week expenditure in the lowest 20% assessed by Minnesota Leisure Time Activity Questionnaire (Richardson et al., 1994). People meeting three or more criteria were classified as frail, those with one or two as pre-frail, and people without any as robust. Also, the level of a sedentary lifestyle was evaluated by the Physical Activity Scale for the Elderly [PASE, (Washburn et al., 1993)].

The neuropsychological evaluation comprised the Montreal Cognitive Assessment [MoCA, (Conti et al., 2015; Santangelo et al., 2015)] to measure the global cognitive level as well as subdomains according to the Uniform Data Set Guidelines (Dodge et al., 2020); the Center for Epidemiologic Studies Depression Scale [CES-D, (Radloff, 1977)] to assess depression symptoms; the Activity of Daily Living Inventory [ADCS-ADL, (Galasko et al., 1997; Reed et al., 2016)] to evaluate the level of autonomy in daily living; the 12-item Short Form Survey [SF12, (Jenkinson et al., 2001)] to measure physical and mental health-related wellbeing; the EuroQoL-5Dimensions-5Levels (Balestroni and Bertolotti, 2012) to measure the quality of life.

#### 2.2.2. Brain structural MRI examination

**Figure 1:**
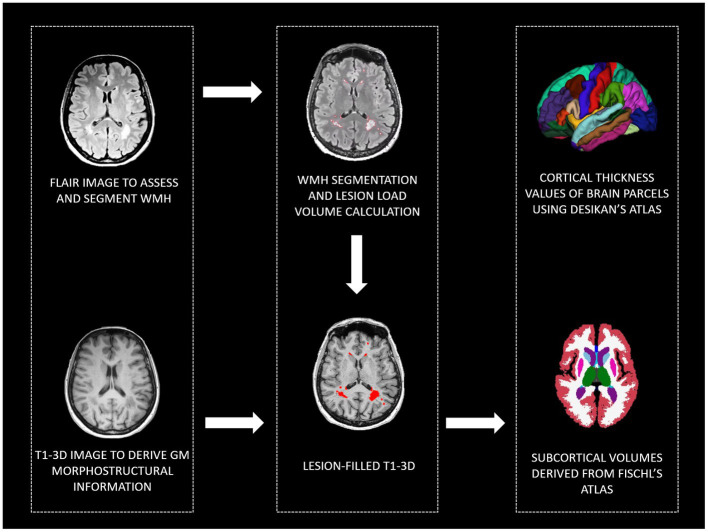
MRI pipeline to derive: WMH, cortical thickness values of brain parcels, and subcortical volumes. WMH, white matter hyperintensities; GM, gray matter, FLAIR, Fluid Attenuated Inversion Recovery; T1-3D, 3D T1 weighted.

To investigate brain morphology, all participants were given a single MRI examination (3 T Siemens PRISMA scanner) including T1-3D (MPRAGE, 0.8 mm^3^, TR/TE: 2,300/3.1, FOV: 256 × 240 mm) to study brain morphometry; FLAIR (0.4 × 0.4 × 1 mm^3^, TR/TE, 5,000/394 ms, FOV, 256 × 230 mm) to assess WMH; T2-weighted to exclude gross brain abnormalities. To extract morphometrical data, after manual segmentation of WMH on FLAIR acquisition, T1-3D images have been lesion filled and analyzed using the recon-all pipeline of Freesurfer software (v. 6.0).^1^ Manual quality controls were performed according to Klapwijk et al. (2019). Manual corrections have been done when necessary. Brain parcellation was performed according to Fischl et al. (2002) and Desikan et al. (2006) atlases to extract brain thickness and subcortical volumes. Moreover, to control for possible cerebrovascular disease involvement, the total volume of the WMH was calculated (see Figure 1 for a graphical representation of the MRI processing steps). Extracted data have been included in second-level statistics, and subcortical volumes and WMH total volume have been normalized using total intracranial volume before entering statistical models.

### 2.3. Statistical analyses

JASP (v. 0.16.1.0; JASP Team 2020) software was utilized for the statistical analyses.

To check the normality distribution of variables, the Kolmogorov–Smirnov normality test, visual inspection of the histogram plot, skewness, and kurtosis were considered, and parametric or non-parametric statistical tests were used as adequate.

Descriptive statistics: to describe the demographics, the clinical and neural profile of participants, the means, medians, interquartile range (IDR), standard deviation, and frequencies were computed. Also, for each subject, the frailty level was considered by computing the frailty score by counting the number of physical criteria of Fried’s frailty phenotype reported (0–5).

Direct group comparisons: to compare demographics, clinical, and neural profiles among Frail, Pre-Frail, and Robust groups, ANCOVA was performed (covariate: age for clinical variables; age and gender for neural variables). The *post hoc* test was computed to identify significant pairwise contrasts between groups. To compare nominal variables among groups, Chi-squared was utilized.

Correlation analyses: the association between frailty, clinical and neural profile (including only regions significantly different between groups at the ANCOVA analyses) was investigated by running partial correlations (covariate: age for clinical variables; age and gender for neural variables). According to Bonferroni’s correction, a value of p threshold of 0.002 for clinical variables and 0.007 for neural variables was set to reduce the rate of false positives.

Regression analyses: clinical and neural indexes correlated with frailty scores were inserted as potential predictors in binary logistic regression. Regardless of the statistical significance, gender, age, and MoCA were inserted into the model. The backward stepwise (Wald) option was used as the selection method. The binary dependent variable was group (pre-frail and frail vs. robust subjects).

Mediation analyses: to test the possible mediator role of clinical variables on the link between neural indexes and frailty, a mediation analysis was performed using the Structural Equation Modeling (SEM) module of JASP software (Biesanz et al., 2010). In detail, we explored: (1) the relationship between neural indexes (predictor, X) and frailty (outcome, Y) (direct effect of X on Y), (2) the relationship between clinical variables (moderator, M) and frailty (Y), (3) the relationship between neural indexes (X) and frailty (Y) following the incorporation of clinical variables (M) (indirect effect). The role of neural indices as moderators was also explored. The standard error estimation was computed (Robust Method option in JASP). All the analyses were adjusted for two covariates: age and sex.

Sample size calculation: the *a-priori* calculation of the study sample size was performed with G*Power software. To identify significant predictors of frailty in a multiple regression model, considering seven predictors in the model, a total sample size of about 90 subjects assured a good power (1-beta error probability = 0.90) with an alpha threshold = 0.05 and an effect size *f*^2^ = 0.15.

## 3. Results

### 3.1. Participants

Overall, a 116 subjects were enrolled in the study as potentially eligible for research participation. Twenty participants were excluded from the analyses (11 did not complete the MRI examination, and 9 presented low-quality MRI data, such as movement artifacts due to a head motion). In total, 96 participants (58 females, mean age ± standard deviation = 75.49 ± 6.62, mean education ± standard deviation = 11.29 ± 3.85) were considered in the analyses. Among these, 17 were classified as frail, 45 were pre-frail, and 34 were robust.

### 3.2. Descriptive statistics: clinical and neuropsychological evaluation

The three groups showed significant differences in years of age (Frail > Pre-frail > Robust), depression level (CES-D, Frail > Pre-frail > Robust), physical health (SF-12, Frail < Pre-frail < Robust), and quality of life (EQ5D5L, Frail < Pre-frail < Robust). Also, the Robust group reported a higher score compared to Pre-frail and Frail group in the global cognitive level (MoCA, MMSE), especially attention (Robust > Frail, Pre-frail), physical activity in daily living (PASE, Robust > Frail, Pre-frail), and mental health (SF-12, Robust > Frail, Pre-frail) (Table 1).

**Table 1:** Subjects’ characteristics and frailty groups’ comparison.

	Frail subjects	Pre-frail subjects	Robust subjects	Test value	*p*-value	η^2^	ω^2^	*post-hoc*
N(%)	17 (18)	45 (47)	34 (35)					
Frailty score (Me, IQR)	3.00, 0.00	1.00, 1.00	0.00, 0.00					
Sex (Ma:Fe)	3:14	18:27	17:17	4.97^^^	0.083			
Age (Me, IQR)	78.00,11.00	75.00,10.00	71.50,6.75	9.58^§^	<0.001	0.17	0.15	F > P > R
Education (Me, IQR)	9.00, 5.00	13.00, 5.00	13.00, 3.00	1.08^§^	0.342			
CES-D (Me, IQR)	24.00, 16.00	13.00,18.00	7.00, 7.00	19.45^§^	<0.001	0.29	0.27	F > P > R
MMSE (Me, IQR)	25.70, 3.90	26.00, 3.00	27.00, 3.55	4.70^§^	0.011	0.09	0.07	R > F,P
MoCA (Me, IQR)	21.36, 5.96	21.83, 3.64	22.94, 3.01	4.71^§^	0.011	0.09	0.07	R > F,P
Memory (0–15)^#^	7.00, 5.00	9.00, 4.00	10.00, 5.00	2.79^§^	0.067			
Executive functions (0–13)^#^	10.00, 4.00	10.00, 4.00	11.50, 1.00	2.95^§^	0.057			
Attention (0–18)^#^	15.00, 6.00	16.00, 3.00	17.00, 2.00	4.03^§^	0.021	0.06	0.20	R > F
Language (0–6)^#^	5.00, 1.00	5.00, 2.00	5.00, 1.00	0.54^§^	0.583			
Visuospatial (0–7)^#^	6.00, 2.00	6.00, 2.00	6.00, 1.00	1.54^§^	0.219			
Orientation (0–6)^#^	6.00, 1.00	6.00, 0.00	6.00, 0.00	0.16^§^	0.851			
PASE (Me, IQR)	64.00, 37.00	71.00, 50.00	108.50, 63.25	5.01^§^	0.009	0.10	0.08	R > F,P
ADCS (Me, IQR)	77.00, 7.00	77.00, 5.00	78.00, 1.00	1.21^§^	0.304			
Basic (0–19)	19.00, 0.00	19.00, 0.00	19.00, 0.00	0.31^§^	0.734			
Communication (0–28)	28.00, 4.00	28.00, 0.00	28.00, 0.00	1.65^§^	0.197			
Domestic (0–20)	19.00, 3.00	20.00, 2.00	20.00, 0.00	2.57^§^	0.082			
Outside (0–13)	13.00, 2.00	13.00, 0.00	13.00, 0.00	0.37^§^	0.692			
SF12 (Me, IQR)								
Physical	37.14, 11.01	44.05, 13.70	46.05, 11.55	5.24^§^	0.007	0.10	0.08	R > P > R
Mental	48.41, 12.14	48.24, 14.97	54.77, 8.56	6.67^§^	0.002	0.12	0.10	R > F,P
EQ5D5L (Me, IQR)								
VAS	50.00, 20.00	70.00, 20.00	72.50,1 8.75	5.02^§^	0.009	0.10	0.08	R > P > R
Index	0.77, 0.20	0.86, 0.12	0.91, 0.07	7.39^§^	0.001	0.14	0.12	R > P > R

ADCS, Activities of Daily Living Inventory; Fe, female subjects; CES-D, Center for Epidemiologic Studies Depression Scale; EQ5D5L, EuroQoL-5Dimensions-5Levels; IQR interquartile range; Me, median; Ma, male; MMSE, Mini-Mental State Examination; MoCA, Montreal Cognitive Assessment.

# derived from the MoCA test according to Dodge et al. (2020); PASE, Physical Activity Scale for the Elderly; SF12, 12-item Short Form Survey.

^ Chi-squared test has been run.

§ ANCOVA test has been run.

A significant difference in Fried’s frailty indicators distribution between the Frail and Pre-frail groups was highlighted, except for weakness. The weakness was the earliest indicator of risk of frailty, while exhaustion represented the symptom that most distinguished Frail from Pre-frail subjects (Table 2). Grouping together Frail and Pre-frails subjects, we found a significant difference between males and females in the frailty score (*t* = −2.29, *p* = 0.026, *d* = 0.61) and in the walking slowness (*t* = −2.48, *p* = 0.016, d = 0.67), which reached a lower level in female than male participants.

**Table 2:** Fried’s frailty indicators frequency in the frail and pre-frail groups.

Fried’s frailty indicator	Frail subjects % of cases on total	Pre-Frail subjects % of cases on total	*χ* ^2^	*p*
Handgrip weakness	82.35% (12/17)	64.44% (29/45)	1.86	0.172
Exhaustion	76.47% (13/17)	24.44% (11/45)	14.07	<0.001
Reduced Activity Level	58.82% (10/17)	22.22% (10/45)	7.56	0.006
Involuntary weight loss	47.06% (8/17)	11.11% (5/45)	9.62	0.002
Walking slowness	47.06% (8/17)	13.33% (6/45)	8.03	0.005

### 3.3. Investigation of neural indexes: MRI examination

The between-group comparison of neural indexes using ANCOVA (Frail, Pre-frail, and Robust groups) highlighted a higher cortical thickness in Robust than in Frail and Pre-frail groups in left parietal–temporal areas, such as postcentral (*F* = 3.97, *p* = 0.022, η^2^ = 0.07, ω^2^ = 0.05), precuneus (*F* = 3.12, *p* = 0.049, η^2^ = 0.06, ω^2^ = 0.04), superior temporal (*F* = 3.50, *p* = 0.034, η^2^ = 0.06, ω^2^ = 0.04), supramarginal (*F* = 4.88, *p* = 0.010, η^2^ = 0.09, ω^2^ = 0.07), and transverse temporal (*F* = 4.04, *p* = 0.021, η^2^ = 0.07, ω^2^ = 0.06) gyri. Also, significant differences (Robust compared to Frail and Pre-frail groups) were found in the right lingual (*F* = 3.24, *p* = 0.044, η^2^ = 0.06, ω^2^ = 0.04) and rostral middle frontal (*F* = 4.43, *p* = 0.015, η^2^ = 0.09, ω^2^ = 0.07) gyri. No significant differences among groups were found in subcortical regions’ volumes and WMH total volume.

### 3.4. Correlation analyses: behavioral and neural indexes associated with frailty score

Concerning demographic variables, age was associated with frailty score (*r* = 0.476, *p*_corr_ = < 0.001). Among behavioral measures, frailty score correlated with CES-D (*r* = 0.634, *p*_corr_ = < 0.001), SF-12_mental health_ (*r* = −0.394, *p*_corr_ = < 0.001), EQ5D5L (*r* = −0.396, *p*_corr_ = < 0.001), and ADCS_domestic activity_ (*r* = −0.325, *p*_corr_ = 0.001). Considering cortical thickness, frailty was associated with left supramarginal (*r* = −0.288, *p*_corr_ = 0.005), and right rostral middle frontal (*r* = −0.292, *p*_corr_ = 0.004) gyri.

No significant correlations were found between the frailty score and subcortical regions’ volumes and between the frailty score and WMH total volume.

### 3.5. Predictors of frailty phenotype and mediation models

Possible predictors of frailty were selected based on correlations’ results and inserted in a binary logistic regression model with a backward method.

The dependent variable was the presence of frailty (Frail and Pre-frail subjects groups versus Robust subjects group). Independent variables considered were: age, gender, MoCA, CES-D, SF-12_mental health_, ADCS_domestic activity_, left supramarginal, and right rostral middle frontal gyrus.

Four models were generated. The best model (Table 3) revealed the predictive role of CES-D, MoCA, and left supramarginal gyrus on the frailty condition (Accuracy = 0.865, AUC = 0.899, Sensitivity = 0.765, Specificity =0.919, Precision = 0.839). Also, the model highlighted a trend of the predictive effect of age and ADCS_domestic activity_ on frailty.

**Table 3:** Binary logistic regression model to test predictors of the frailty syndrome.

Predictors	β	Odds ratio	Wald	*p*	95% CI	
					Lower bound	Upper bound
CES-D	0.13	1.138	10.51	0.001	0.05	0.21
Left supramarginal gyrus	−5.49	0.004	3.97	0.046	−10.90	−0.09
MoCA	−0.23	0.792	4.00	0.046	−0.46	−0.00
ADCS_domestic activity_	−0.74	0.477	3.67	0.055	−1.50	0.02
Age	0.09	1.094	2.98	0.084	−0.01	0.19
Intercept	25.40	0.00	4.42	0.036	1.72	49.08

CES-D, Center for Epidemiologic Studies Depression Scale; MoCA, Montreal Cognitive Assessment; ADCS, Activities of Daily Living Inventory; CI, Confidence Interval.

**Figure 2:**
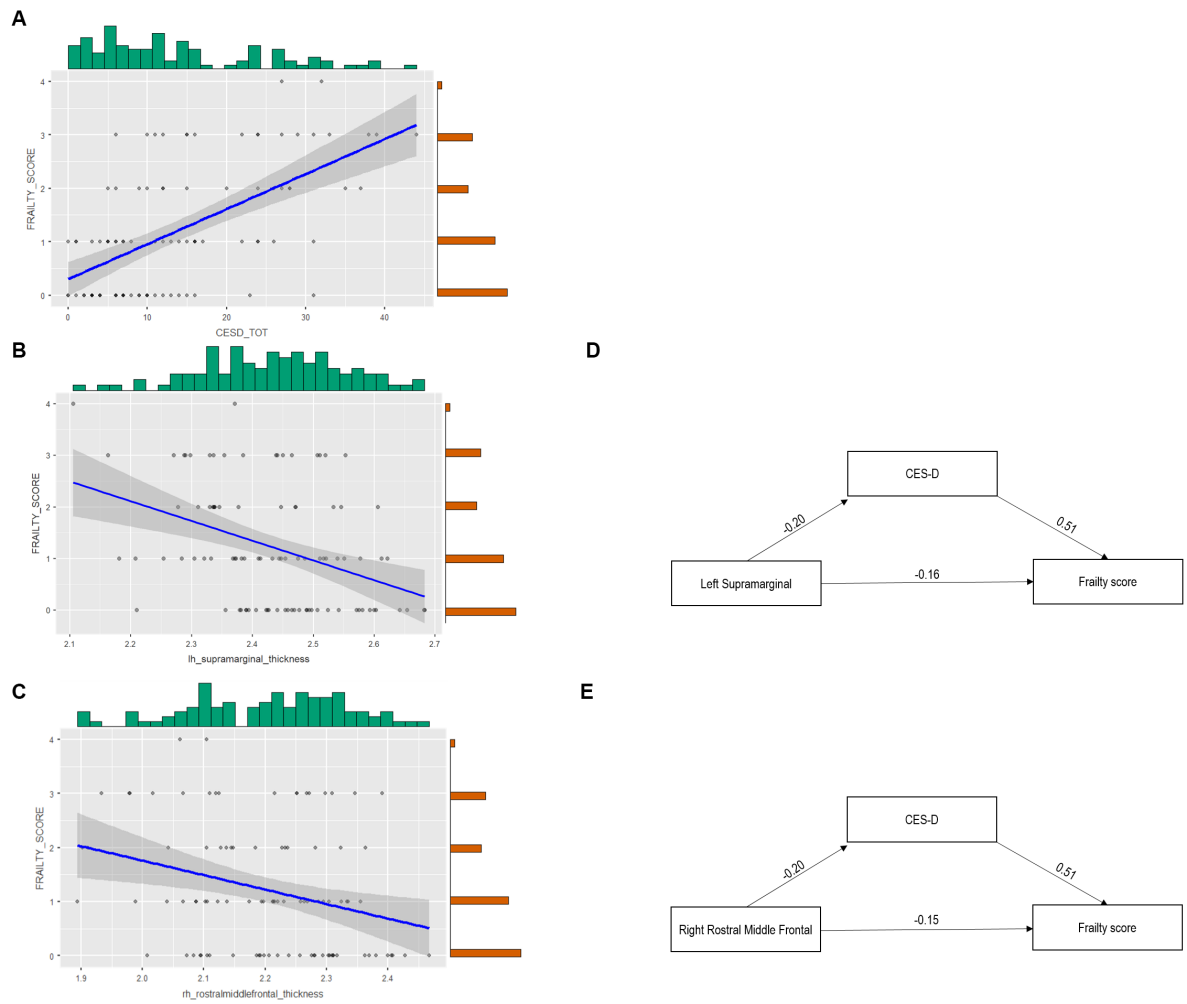
The complex link between cortical thickness, depression, and frailty. Panel **(A)** reports the Scatter plot depicting the link between frailty Score and CES-D. Panel **(B)** depicts the scatter plot of left supramarginal gyrus thickness and the frailty score. Panel **(C)** depicts the scatter plot of right rostral middle frontal gyrus thickness and the frailty score (light gray dots indicate that only one subject reported a certain relationship between variables, while dark gray dots suggest that more than one subject reported the relationship). Panel **(D)** reports the Mediation of CES-D on the link between left supramarginal thickness and the frailty score. Panel **(E)** shows the Mediation of CES-D on the link between right rostral middle frontal gyrus thickness and the frailty score.

The mediation model revealed a full mediation of depression on the link between the neural index and frailty. Specifically, Table 4 shows the significant mediation role of CES-D on the link between frailty and both the left supramarginal and the right rostral middle frontal gyri (Figure 2).

**Table 4:** Mediation models testing the role of the left supramarginal and right rostral middle frontal gyri’s thickness on the link between depression and frailty.

		Estimate	SE	*z*-value	*p*	95% CI	
						Lower bound	Lower bound
*Mediation role of left supramarginal gyrus*							
Direct effect	L supramarginal ➔ frailty score	−0.16	0.09	−1.80	0.072	−0.33	0.01
Indirect effect	L supramarginal ➔ CES-D ➔ frailty score	−0.10	0.05	−2.18	0.029	−0.20	−0.01
Total effect	L supramarginal ➔ frailty score	−0.26	0.09	−2.82	0.005	−0.45	−0.08
*Mediation role of right rostral middle frontal gyrus*							
Direct effect	R rostral middle frontal ➔ frailty score	−1.34	0.79	−1.69	0.091	−2.89	0.21
Indirect effect	R rostral middle frontal ➔ CES-D ➔ frailty score	−0.98	0.45	−2.19	0.029	−1.85	−0.10
Total effect	R rostral middle frontal ➔ frailty score	−2.32	0.86	−2.69	0.007	−4.01	−0.63

CES-D, Center for Epidemiologic Studies Depression Scale; CI, Confidence Interval; L, left; R = right; SE, standard error.

The same analyses have been run considering the mediating role of MoCA, and no statistically significant direct and/or indirect effects have been revealed.

Supplementary material report two additional mediation models testing the effect of the left supramaginal gyrus and of the right rostral middle frontal gyrus on the link between CES-D and frailty. Results yielded no statistically significant effects (see Supplementary Tables S1, S2).

## 4. Discussion

The present study aimed to disentangle the complex relationship between frailty, clinical profile, and neural pattern, investigating each component’s predicting and mediating role. The findings relied on a representative cohort of the general population of older people. As expected, frail people were a limited percentage of our study’s total cohort of subjects; frailty subjects were older (Canevelli et al., 2015), with a prevalence of females (Hanlon et al., 2018; Williams et al., 2018). Frail and pre-frail subjects presented more depressive symptoms, lower physical activity, reduced quality of life and well-being, and lower global cognitive level than not-frail people.

The main result of the present study was the finding of a full mediation of depression on the link between frailty and brain cortical thickness in the supramarginal and rostral middle frontal gyri. Interestingly, in this link, no mediating role was found for the cognitive level. This datum suggests a twofold role of depression: a central role in the prediction of the risk of developing a frailty phenotype but also an explanatory role in the link between the neural integrity (cortical thickness) and the risk of developing a frailty phenotype. While the role of depression in the risk of developing Frailty is well acknowledged in some cohort and meta-analysis studies (Mezuk et al., 2012; Brown et al., 2014; Soysal et al., 2017; Chu et al., 2019), the second aspect linking depression to brain integrity and fragility is unprecedented.

Previous findings have demonstrated the strict relationship between frailty and depression, the latter being highly prevalent in both community dwelling and nursing home older adults (Giovannini et al., 2020). A recent meta-analysis involving 84,351 older adults (Chu et al., 2019) showed how older adults with depression were more prone to frailty than those without depression; this was especially true in men. Moreover, this risk was rated as high as an 80% probability of older adults with depression being frail (Pegorari and Tavares, 2014). Finally, Brown et al. (2014) found that the concurrence of specific characteristics of frailty, such as fatigue and slow gait speed, with depression in older adults was associated with an increased risk of death; this association was stronger for older depressed women than men (Isernia et al., 2023). In fact, previous studies showed that depressive symptomatology is an early risk factor for frailty in women, where the increment of depression is evident already in pre-frail phenotype (Isernia et al., 2023). Altogether, the herein presented data together with the literature suggest the importance of treating depression in the cure and prevention of frailty. The role of depression in the frailty phenotype can be interpreted in many ways. One possibility is that the loss of interest and engagement in daily life activities (Vaughan et al., 2015; Gale et al., 2018) facilitates the risk of low physical activity, with consequent loss of physical capacities and increased risk for falls, and weight loss, all of which may increase the risk for frailty (Lohman et al., 2022). In this line, a recent meta-analysis showed how depression and frailty in older adults are each associated with an increased prevalence and incidence of the other and represent a risk factor for the development of the other (Soysal et al., 2017), thus pointing to a reciprocal interaction between the two conditions. According to this view, our results favor considering the reciprocal interaction between psychological and physical aspects of health for clinical care, supporting the notion that “mental health becomes health” (Schnittker, 2005).

As stated above, the novelty of this study relies on the finding of the mediating role of depression in the link between brain integrity and fragility involving two brain areas: the left supramarginal and the right rostral middle frontal gyri. It should be noted that only the left supramarginal gyrus was a significant predictor of frailty, suggesting a causal role in fragility.

Previous studies separately investigated the role of the left supramarginal gyrus in frailty syndrome and depression. Evidence from neuroimaging techniques (Suárez-Méndez et al., 2020) showed reduced functional connectivity of this area with frontal motor control regions in frailty subjects, suggesting a role in motor impairments in this population. Moreover, the left supramarginal gyrus, a multimodal/multisensory area, has been associated with an integrative role in the perception of the position and movement of own body in space (Proske and Gandevia, 2012). Finally, this area has been involved in emotion processing and regulation in patients with psychiatric disorders (Madeira et al., 2020), and an aberrant pattern of activation of this gyrus at rest has been highlighted in bipolar and major depression patients (Gong et al., 2020).

The herein presented results are in agreement with the above-reported evidence from literature suggesting how the relation of the left supramarginal gyrus thickness with frailty is mediated by depression through mechanisms involving reduced proprioception, movement guidance, and emotion regulation.

Regarding the specific left-lateralized contribution of the supramarginal gyrus, we registered an asymmetrical gray matter reduction in thickness of frail and pre-frail subjects, prevalent in the left hemisphere, that is peculiar to aging and people at risk of neurodegenerative conditions, showing the so-called “left hemisphere susceptibility” (Shi et al., 2009; Donix et al., 2013; Cabinio et al., 2018; Yang et al., 2019). The greater dependence on left hemisphere processing in older adults is also supported by the HAROLD model (“hemispheric asymmetry reduction in older adults”), which describes changes in functional recruitment of brain hemispheres in aging due to a global reorganization of neurocognitive networks as well as regional neural changes.

Concerning the role of depression on the link between frailty and the right rostral middle frontal gyrus, it is noteworthy that the latter is part of the dorsolateral prefrontal cortex, which is known to be implicated in executive functions and in late life depression (Aizenstein et al., 2009). Specifically, an altered functioning in the executive control circuit has been observed in patients with major depression, mainly related to the right rostral middle frontal gyrus hypo-activity, which may be amenable to treatment.

Another finding in this study relates to the prevalence of frailty indicators in frail and pre-frail subjects, showing how handgrip weakness was the predominant symptom in both frail and pre-frail groups. At the same time, exhaustion was the indicator that most distinguished frail from pre-frail people. This is in line with previous research that reported handgrip weakness [i.e., Women’s Health and Ageing Study II (Xue et al., 2008)] and exhaustion [i.e., inCHIANTI study (Stenholm et al., 2019)] as the earliest component of frailty. Thus, handgrip weakness may detect people at risk of frailty syndrome, while exhaustion may represent the leading indicator of actual frailty occurrence.

Some study limitations must be considered: our sample size is small, and the results need to be confirmed with a broader sample to assure generalizability. Also, further research adopting a longitudinal design may verify the findings related to the mediation model. Moreover, we restricted our model to investigate the link between neural patterns, depression, cognitive impairment, and frailty. However, additional significant variables, such as the muscle’s integrity, nutritional lifestyle, and biomolecular data, should be considered.

Despite these caveats, the clinical implications of this study are significant. Clinicians should focus on depression and handgrip weakness, and interventions to prevent and reverse the frailty syndrome may act against muscle strength loss (Giovannini et al., 2021) isolation and disengagement in daily living. Especially differently to the current treatments targeted for frailty people, mainly operating on physical enhancement, social inclusion, and engagement should be considered. Accordingly, non-pharmacological treatments stimulating physical, emotional, and social processes, such as dance-based rehabilitation therapy (Meekums et al., 2015; Millman et al., 2021) or group activities (Savazzi et al., 2020), may show potential benefits for the frailty population.

## 5. Conclusion

In conclusion, this research supports the notion of frailty as a complex clinical entity in which depression mediates the association between brain integrity (supramarginal gyrus thickness) and Frailty. Moreover, our data showed how handgrip weakness is a crucial indicator of frailty. However, future contributions may confirm these results by adopting a longitudinal design and testing the effectiveness of rehabilitative interventions for people with frailty acting on depressive symptoms.

## Data availability statement

The raw data supporting the conclusions of this article will be made available by the authors, without undue reservation.

## Ethics statement

The study involving humans was approved by Don Gnocchi Foundation Ethics Committee. The studies were conducted in accordance with the local legislation and institutional requirements. The participants provided their written informed consent to participate in this study.

## Author contributions

SI: Formal analysis, Investigation, Writing – original draft. VB: Conceptualization, Methodology, Supervision, Writing – original draft, Writing – review & editing. GB: Conceptualization, Validation, Writing – review & editing. MCab: Data curation, Writing – review & editing. PC: Investigation, Writing – review & editing. FR: Data curation, Writing – original draft. MCaz: Investigation, Writing – review & editing. FBl: Conceptualization, Funding acquisition, Writing – review & editing. CB: Investigation, Writing – review & editing. FG: Data curation, Writing – review & editing. SS: Supervision, Writing – review & editing. MCle: Supervision, Writing – review & editing. FBa: Funding acquisition, Supervision, Writing – original draft, Writing – review & editing.

## Footnotes

^1^ https://surfer.nmr.mgh.harvard.edu/

## References

[ref1] Aizenstein H. J. Butters M. A. Wu M. Mazurkewicz L. M. Stenger V. A. Gianaros P. J.. (2009). Altered functioning of the executive control circuit in late-life depression: episodic and persistent phenomena. Am. J. Geriatr. Psych. Off. J. Am. Assoc. Geriatr. Psych. 17, 30–42. doi: 10.1097/JGP.0b013e31817b60af PMC2626170 19001356

[ref2] Avila-Funes J. A. Amieva H. Barberger-Gateau P. Le Goff M. Raoux N. Ritchie K.. (2009). Cognitive impairment improves the predictive validity of the phenotype of frailty for adverse health outcomes: the three-city study. J. Am. Geriatr. Soc. 57, 453–461. doi: 10.1111/j.1532-5415.2008.02136.x, PMID: 19245415

[ref3] Avila-Funes J. A. Carcaillon L. Helmer C. Carrière I. Ritchie K. Rouaud O.. (2012). Is frailty a prodromal stage of vascular dementia? Results from the Three-City study. J. Am. Geriatr. Soc. 60, 1708–1712. doi: 10.1111/j.1532-5415.2012.04142.x, PMID: 22985143

[ref4] Avila-Funes J. A. Pelletier A. Meillon C. Catheline G. Periot O. Trevin O. F. I.. (2017). Vascular cerebral damage in frail older adults: the AMImage study. J. Gerontol. A Biol. Sci. Med. Sci. 72, 971–977. doi: 10.1093/gerona/glw347, PMID: 28329104

[ref5] Balestroni G. Bertolotti G. (2012). EuroQol-5D (EQ-5D): an instrument for measuring quality of life. Monaldi Arch. Chest Dis. 78, 155–159. doi: 10.4081/monaldi.2012.121 23614330

[ref6] Bandeen-Roche K. Xue Q. L. Ferrucci L. Walston J. Guralnik J. M. Chaves P.. (2006). Phenotype of frailty: characterization in the women's health and aging studies. J. Gerontol. A Biol. Sci. Med. Sci. 61, 262–266. doi: 10.1093/gerona/61.3.262 16567375

[ref7] Biesanz J. C. Falk C. F. Savalei V. (2010). Assessing mediational models: testing and interval estimation for indirect effects. Multivar. Behav. Res. 45, 661–701. doi: 10.1080/00273171.2010.498292 26735714

[ref8] Bohannon R. W. (1997). Comfortable and maximum walking speed of adults aged 20-79 years: reference values and determinants. Age Ageing 26, 15–19. doi: 10.1093/ageing/26.1.15, PMID: 9143432

[ref9] Brown P. J. Roose S. P. Fieo R. Liu X. Rantanen T. Sneed J. R.. (2014). Frailty and depression in older adults: a high-risk clinical population. Am. J. Geriatr. Psychiatry 22, 1083–1095. doi: 10.1016/j.jagp.2013.04.010, PMID: 23973252 PMC3930630

[ref10] Buigues C. Padilla-Sánchez C. Garrido J. F. Navarro-Martínez R. Ruiz-Ros V. Cauli O. (2015). The relationship between depression and frailty syndrome: a systematic review. Aging Ment. Health 19, 762–772. doi: 10.1080/13607863.2014.967174 25319638

[ref11] Bunce D. Batterham P. J. Mackinnon A. J. (2019). Long-term associations between physical frailty and performance in specific cognitive domains. J. Gerontol. B Psychol. Sci. Soc. Sci. 74, 919–926. doi: 10.1093/geronb/gbx177 29401240

[ref12] Bunt S. Steverink N. Olthof J. van der Schans C. P. Hobbelen J. S. M. (2017). Social frailty in older adults: a scoping review. Eur. J. Ageing 14, 323–334. doi: 10.1007/s10433-017-0414-7 28936141 PMC5587459

[ref13] Cabinio M. Saresella M. Piancone F. LaRosa F. Marventano I. Guerini F. R.. (2018). Association between hippocampal shape, Neuroinflammation, and cognitive decline in Alzheimer's disease. J. Alzheimers Dis. 66, 1131–1144. doi: 10.3233/jad-180250, PMID: 30400090

[ref14] Canevelli M. Cesari M. van Kan G. A. (2015). Frailty and cognitive decline: how do they relate? Curr. Opin. Clin. Nutr. Metab. Care 18, 43–50. doi: 10.1097/mco.0000000000000133 25405314

[ref15] Chang S. S. Weiss C. O. Xue Q. L. Fried L. P. (2010). Patterns of comorbid inflammatory diseases in frail older women: the Women's health and aging studies I and II. J. Gerontol. A Biol. Sci. Med. Sci. 65, 407–413. doi: 10.1093/gerona/glp181, PMID: 19933749 PMC3004772

[ref16] Cheung J. T. K. Yu R. Wu Z. Wong S. Y. S. Woo J. (2018). Geriatric syndromes, multimorbidity, and disability overlap and increase healthcare use among older Chinese. BMC Geriatr. 18:147. doi: 10.1186/s12877-018-0840-1, PMID: 29940868 PMC6019236

[ref17] Chu W. Chang S. F. Ho H. Y. Lin H. C. (2019). The relationship between depression and frailty in community-dwelling older people: a systematic review and Meta-analysis of 84,351 older adults. J. Nurs. Scholarsh. 51, 547–559. doi: 10.1111/jnu.12501, PMID: 31328878

[ref18] Clegg A. Young J. Iliffe S. Rikkert M. O. Rockwood K. (2013). Frailty in elderly people. Lancet 381, 752–762. doi: 10.1016/s0140-6736(12)62167-9, PMID: 23395245 PMC4098658

[ref19] Conti S. Bonazzi S. Laiacona M. Masina M. Coralli M. V. (2015). Montreal cognitive Assessmen (MoCA)-Italian version: regression based norms and equivalent scores. Neurol. Sci. 36, 209–214. doi: 10.1007/s10072-014-1921-3 25139107

[ref20] Del Brutto O. H. Mera R. M. Cagino K. Fanning K. D. Milla-Martinez M. F. Nieves J. L.. (2017). Neuroimaging signatures of frailty: a population-based study in community-dwelling older adults (the Atahualpa project). Geriatr Gerontol Int 17, 270–276. doi: 10.1111/ggi.12708, PMID: 26790541

[ref21] Dent E. Martin F. C. Bergman H. Woo J. Romero-Ortuno R. Walston J. D. (2019). Management of frailty: opportunities, challenges, and future directions. Lancet 394, 1376–1386. doi: 10.1016/s0140-6736(19)31785-4, PMID: 31609229

[ref22] Desikan R. S. Ségonne F. Fischl B. Quinn B. T. Dickerson B. C. Blacker D.. (2006). An automated labeling system for subdividing the human cerebral cortex on MRI scans into gyral based regions of interest. NeuroImage 31, 968–980. doi: 10.1016/j.neuroimage.2006.01.021 16530430

[ref9001] Dodge H. H. Goldstein F. C. Wakim N. I. Gefen T. Teylan M. Chan K. C. G.. (2020). Differentiating among stages of cognitive impairment in aging: Version 3 of the Uniform Data Set (UDS) neuropsychological test battery and MoCA index scores. Alzheimer’s Dement. 6:e12103. doi: 10.1002/trc2.12103 PMC7683960 33283037

[ref23] Donix M. Burggren A. C. Scharf M. Marschner K. Suthana N. A. Siddarth P.. (2013). APOE associated hemispheric asymmetry of entorhinal cortical thickness in aging and Alzheimer's disease. Psychiatry Res. 214, 212–220. doi: 10.1016/j.pscychresns.2013.09.006, PMID: 24080518 PMC3851589

[ref24] Ducca E. L. Gomez G. T. Palta P. Sullivan K. J. Jack C. R. Knopman D. S.. (2023). Physical frailty and brain white matter abnormalities: the atherosclerosis risk in communities study. J. Gerontol. A Biol. Sci. Med. Sci. 78, 357–364. doi: 10.1093/gerona/glac111, PMID: 35596270 PMC9951053

[ref25] Feng L. Zin Nyunt M. S. Gao Q. Yap K. B. Ng T. P. (2017). Cognitive frailty and adverse health outcomes: findings from the Singapore longitudinal ageing studies (SLAS). J. Am. Med. Dir. Assoc. 18, 252–258. doi: 10.1016/j.jamda.2016.09.015, PMID: 27838339

[ref26] Fischl B. Salat D. H. Busa E. Albert M. Dieterich M. Haselgrove C.. (2002). Whole brain segmentation: automated labeling of neuroanatomical structures in the human brain. Neuron 33, 341–355. doi: 10.1016/s0896-6273(02)00569-x 11832223

[ref27] Fried L. P. Tangen C. M. Walston J. Newman A. B. Hirsch C. Gottdiener J.. (2001). Frailty in older adults: evidence for a phenotype. J. Gerontol. A Biol. Sci. Med. Sci. 56, M146–M156. doi: 10.1093/gerona/56.3.m146 11253156

[ref28] Galasko D. Bennett D. Sano M. Ernesto C. Thomas R. Grundman M.. (1997). An inventory to assess activities of daily living for clinical trials in Alzheimerʼs disease. Alzheimer Dis. Assoc. Disord. 11, 33–39. doi: 10.1097/00002093-199700112-00005 9236950

[ref29] Gale C. R. Westbury L. Cooper C. (2018). Social isolation and loneliness as risk factors for the progression of frailty: the English longitudinal study of ageing. Age Ageing 47, 392–397. doi: 10.1093/ageing/afx188, PMID: 29309502 PMC5920346

[ref30] Giovannini S. Coraci D. Brau F. Galluzzo V. Loreti C. Caliandro P.. (2021). Neuropathic pain in the elderly. Diagnostics 11:613. doi: 10.3390/diagnostics11040613, PMID: 33808121 PMC8066049

[ref31] Giovannini S. Onder G. van der Roest H. G. Topinkova E. Gindin J. Cipriani M. C.. (2020). Use of antidepressant medications among older adults in European long-term care facilities: a cross-sectional analysis from the SHELTER study. BMC Geriatr. 20:310. doi: 10.1186/s12877-020-01730-5, PMID: 32854659 PMC7457305

[ref32] Gong J. Wang J. Qiu S. Chen P. Luo Z. Huang L.. (2020). Common and distinct patterns of intrinsic brain activity alterations in major depression and bipolar disorder: voxel-based meta-analysis. Transl. Psychiatry 10:353. doi: 10.1038/s41398-020-01036-5, PMID: 33077728 PMC7573621

[ref33] Hanlon P. Nicholl B. I. Jani B. D. Lee D. McQueenie R. Mair F. S. (2018). Frailty and pre-frailty in middle-aged and older adults and its association with multimorbidity and mortality: a prospective analysis of 493 737 UK biobank participants. Lancet Public Health 3, e323–e332. doi: 10.1016/s2468-2667(18)30091-4 29908859 PMC6028743

[ref34] Isernia S. Cazzoli M. Baglio G. Cabinio M. Rossetto F. Giunco F.. (2023). Differential roles of neural integrity, physical activity and depression in frailty: sex-related differences. Brain Sci. 13:950. doi: 10.3390/brainsci13060950, PMID: 37371428 PMC10296427

[ref35] Jenkinson C. Chandola T. Coulter A. Bruster S. (2001). An assessment of the construct validity of the SF-12 summary scores across ethnic groups. J. Public Health Med. 23, 187–194. doi: 10.1093/pubmed/23.3.187 11585190

[ref36] KelaiditiE.CesariM.CanevelliM.van KanG. A.OussetP. J.Gillette-GuyonnetS.. (2013). Cognitive frailty: rational and definition from an (I.A.N.A./I.A.G.G.) international consensus group. J. Nutr. Health Aging 17, 726–734. doi: 10.1007/s12603-013-0367-224154642

[ref37] Klapwijk E. T. van de Kamp F. van der Meulen M. Peters S. Wierenga L. M. (2019). Qoala-T: a supervised-learning tool for quality control of FreeSurfer segmented MRI data. NeuroImage 189, 116–129. doi: 10.1016/j.neuroimage.2019.01.014 30633965

[ref38] Lohman M. C. Mezuk B. Fairchild A. J. Resciniti N. V. Merchant A. T. (2022). The role of frailty in the association between depression and fall risk among older adults. Aging Ment. Health 26, 1805–1812. doi: 10.1080/13607863.2021.1950616, PMID: 35993919 PMC9395731

[ref39] López-Sanz D. Suárez-Méndez I. Bernabé R. Pasquín N. Rodríguez-Mañas L. Maestú F.. (2018). Scoping review of neuroimaging studies investigating frailty and frailty components. Front. Med. 5:284. doi: 10.3389/fmed.2018.00284, PMID: 30349819 PMC6186819

[ref40] Lu W. H. de Souto B. P. Rolland Y. Rodríguez-Mañas L. Bouyahia A. Fischer C.. (2020). Cross-sectional and prospective associations between cerebral cortical thickness and frailty in older adults. Exp. Gerontol. 139:111018. doi: 10.1016/j.exger.2020.111018, PMID: 32663588

[ref41] Madeira N. Duarte J. V. Martins R. Costa G. N. Macedo A. Castelo-Branco M. (2020). Morphometry and gyrification in bipolar disorder and schizophrenia: a comparative MRI study. Neuroimage Clin. 26:102220. doi: 10.1016/j.nicl.2020.102220, PMID: 32146321 PMC7063231

[ref42] Maltais M. de Souto B. P. Perus L. Mangin J. F. Grigis A. Chupin M.. (2020). Prospective associations between diffusion tensor imaging parameters and frailty in older adults. J. Am. Geriatr. Soc. 68, 1050–1055. doi: 10.1111/jgs.16343 31981370

[ref43] Meekums B. Karkou V. Nelson E. A. (2015). Dance movement therapy for depression. Cochrane Database Syst. Rev. 2016:CD009895. doi: 10.1002/14651858.CD009895.pub2, PMID: 25695871 PMC8928931

[ref44] Mezuk B. Edwards L. Lohman M. Choi M. Lapane K. (2012). Depression and frailty in later life: a synthetic review. Int. J. Geriatr. Psychiatry 27, 879–892. doi: 10.1002/gps.2807 21984056 PMC3276735

[ref45] Millman L. S. M. Terhune D. B. Hunter E. C. M. Orgs G. (2021). Towards a neurocognitive approach to dance movement therapy for mental health: a systematic review. Clin. Psychol. Psychother. 28, 24–38. doi: 10.1002/cpp.2490 32539160

[ref46] Mitnitski A. B. Mogilner A. J. Rockwood K. (2001). Accumulation of deficits as a proxy measure of aging. ScientificWorldJournal 1, 323–336. doi: 10.1100/tsw.2001.58, PMID: 12806071 PMC6084020

[ref47] Panza F. Seripa D. Solfrizzi V. Tortelli R. Greco A. Pilotto A.. (2015a). Targeting cognitive frailty: clinical and neurobiological roadmap for a single complex phenotype. J. Alzheimers Dis. 47, 793–813. doi: 10.3233/jad-150358, PMID: 26401761

[ref48] Panza F. Solfrizzi V. Barulli M. R. Santamato A. Seripa D. Pilotto A.. (2015b). Cognitive frailty: a systematic review of epidemiological and neurobiological evidence of an age-related clinical condition. Rejuvenation Res. 18, 389–412. doi: 10.1089/rej.2014.1637, PMID: 25808052

[ref49] Pegorari M. S. Tavares D. M. (2014). Factors associated with the frailty syndrome in elderly individuals living in the urban area. Rev. Lat. Am. Enfermagem 22, 874–882. doi: 10.1590/0104-1169.0213.2493, PMID: 25493685 PMC4292678

[ref50] Pezzotti P. Scalmana S. Mastromattei A. Di Lallo D. (2008). The accuracy of the MMSE in detecting cognitive impairment when administered by general practitioners: a prospective observational study. BMC Fam. Pract. 9:29. doi: 10.1186/1471-2296-9-29 18477390 PMC2426687

[ref51] Proske U. Gandevia S. C. (2012). The proprioceptive senses: their roles in signaling body shape, body position and movement, and muscle force. Physiol. Rev. 92, 1651–1697. doi: 10.1152/physrev.00048.2011, PMID: 23073629

[ref52] Radloff L. S. (1977). The CES-D scale: a self-report depression scale for research in the general population. Appl. Psychol. Meas. 1, 385–401. doi: 10.1177/014662167700100306

[ref53] Reed C. Mark B. Vellas B. Andrews J. S. Argimon J. M. Bruno G.. (2016). Identifying factors of activities of daily living important for cost and caregiver outcomes in Alzheimer's disease. Int. Psychogeriatr. 28, 247–259. doi: 10.1017/S1041610215001349 26307191

[ref54] Richardson M. T. Leon A. S. Jacobs D. R. Ainsworth B. E. Serfass R. (1994). Comprehensive evaluation of the Minnesota leisure time physical activity questionnaire. J. Clin. Epidemiol. 47, 271–281. doi: 10.1016/0895-4356(94)90008-6 8138837

[ref55] Rockwood K. Mitnitski A. (2007). Frailty in relation to the accumulation of deficits. J. Gerontol. A Biol. Sci. Med. Sci. 62, 722–727. doi: 10.1093/gerona/62.7.722 17634318

[ref56] Ruan Q. Yu Z. Chen M. Bao Z. Li J. He W. (2015). Cognitive frailty, a novel target for the prevention of elderly dependency. Ageing Res. Rev. 20, 1–10. doi: 10.1016/j.arr.2014.12.004 25555677

[ref57] Santangelo G. Siciliano M. Pedone R. Vitale C. Falco F. Bisogno R.. (2015). Normative data for the Montreal cognitive assessment in an Italian population sample. Neurol. Sci. 36, 585–591. doi: 10.1007/s10072-014-1995-y 25380622

[ref58] Savazzi F. Isernia S. Farina E. Fioravanti R. D'Amico A. Saibene F. L.. (2020). "art, colors, and emotions" treatment (ACE-t): a pilot study on the efficacy of an art-based intervention for people with Alzheimer's disease. Front. Psychol. 11:1467. doi: 10.3389/fpsyg.2020.01467 32765343 PMC7378782

[ref59] Schnittker J. (2005). When mental health becomes health: age and the shifting meaning of self-evaluations of general health. Milbank Q. 83, 397–423. doi: 10.1111/j.1468-0009.2005.00407.x, PMID: 16201998 PMC2690150

[ref60] Shi F. Liu B. Zhou Y. Yu C. Jiang T. (2009). Hippocampal volume and asymmetry in mild cognitive impairment and Alzheimer's disease: Meta-analyses of MRI studies. Hippocampus 19, 1055–1064. doi: 10.1002/hipo.20573, PMID: 19309039

[ref61] Siejka T. P. Srikanth V. K. Hubbard R. E. Moran C. Beare R. Wood A.. (2020). White matter Hyperintensities and the progression of frailty-the Tasmanian study of cognition and gait. J. Gerontol. A Biol. Sci. Med. Sci. 75, 1545–1550. doi: 10.1093/gerona/glaa024, PMID: 31956917

[ref62] Soysal P. Veronese N. Thompson T. Kahl K. G. Fernandes B. S. Prina A. M.. (2017). Relationship between depression and frailty in older adults: a systematic review and meta-analysis. Ageing Res. Rev. 36, 78–87. doi: 10.1016/j.arr.2017.03.005, PMID: 28366616

[ref63] Stenholm S. Ferrucci L. Vahtera J. Hoogendijk E. O. Huisman M. Pentti J.. (2019). Natural course of frailty components in people who develop frailty syndrome: evidence from two cohort studies. J. Gerontol. A Biol. Sci. Med. Sci. 74, 667–674. doi: 10.1093/gerona/gly132, PMID: 30084927 PMC6477647

[ref64] Sternberg S. A. Wershof Schwartz A. Karunananthan S. Bergman H. Mark C. A. (2011). The identification of frailty: a systematic literature review. J. Am. Geriatr. Soc. 59, 2129–2138. doi: 10.1111/j.1532-5415.2011.03597.x 22091630

[ref65] Suárez-Méndez I. Doval S. Walter S. Pasquín N. Bernabé R. Gallo E. C.. (2020). Functional connectivity disruption in frail older adults without global cognitive deficits. Front. Med. 7:322. doi: 10.3389/fmed.2020.00322 PMC7360673 32733905

[ref66] Sugimoto T. Sakurai T. Ono R. Kimura A. Saji N. Niida S.. (2018). Epidemiological and clinical significance of cognitive frailty: a mini review. Ageing Res. Rev. 44, 1–7. doi: 10.1016/j.arr.2018.03.002 29544875

[ref67] Tian Q. Williams O. A. Landman B. A. Resnick S. M. Ferrucci L. (2020). Microstructural neuroimaging of frailty in cognitively Normal older adults. Front. Med. 7:546344. doi: 10.3389/fmed.2020.546344 PMC7645067 33195297

[ref68] Vaughan L. Corbin A. L. Goveas J. S. (2015). Depression and frailty in later life: a systematic review. Clin. Interv. Aging 10, 1947–1958. doi: 10.2147/cia.s69632 26719681 PMC4687619

[ref69] Washburn R. A. Smith K. W. Jette A. M. Janney C. A. (1993). The physical activity scale for the elderly (PASE): development and evaluation. J. Clin. Epidemiol. 46, 153–162. doi: 10.1016/0895-4356(93)90053-4 8437031

[ref70] Williams B. Jalilianhasanpour R. Matin N. Fricchione G. L. Sepulcre J. Keshavan M. S.. (2018). Individual differences in corticolimbic structural profiles linked to insecure attachment and coping styles in motor functional neurological disorders. J. Psychiatr. Res. 102, 230–237. doi: 10.1016/j.jpsychires.2018.04.006, PMID: 29702433 PMC6005758

[ref71] Xue Q. L. (2011). The frailty syndrome: definition and natural history. Clin. Geriatr. Med. 27, 1–15. doi: 10.1016/j.cger.2010.08.009, PMID: 21093718 PMC3028599

[ref72] Xue Q. L. Bandeen-Roche K. Varadhan R. Zhou J. Fried L. P. (2008). Initial manifestations of frailty criteria and the development of frailty phenotype in the Women's health and aging study II. J. Gerontol. A Biol. Sci. Med. Sci. 63, 984–990. doi: 10.1093/gerona/63.9.984, PMID: 18840805

[ref73] Yang H. Xu H. Li Q. Jin Y. Jiang W. Wang J.. (2019). Study of brain morphology change in Alzheimer's disease and amnestic mild cognitive impairment compared with normal controls. Gen. Psych. 32:e100005. doi: 10.1136/gpsych-2018-100005, PMID: 31179429 PMC6551438

