# Supplementary material for: The key role of depression and supramarginal gyrus in frailty: a cross-sectional study
Source: Front Aging Neurosci. 2023 Nov 9;15:1292417. doi: 10.3389/fnagi.2023.1292417 (PMC10665836; doi:10.3389/fnagi.2023.1292417)
Supplement: Supplementary file 1 [file Table_1.docx]

The key role of depression and supramarginal gyrus in frailty: a cross-sectional study

Sara Isernia^1^, Valeria Blasi^1^*, Gisella Baglio^1^, Monia Cabinio^1^, Pietro Cecconi^1^, Federica Rossetto^1^, Marta Cazzoli^1^, Francesco Blasi^2^, Chiara Bruckmann^2^, Fabrizio Giunco^1^, Sandro Sorbi^1^, Mario Clerici^1,3^, Francesca Baglio^1^

**Supplementary Materials**

**S1. Mediation model testing the mediation effect of left supramarginal gyrus on the link between CES-D and Frailty score.**

The mediation model revealed no mediation of the left supramarginal thickness on the link between depression and frailty (see table S1).

|  | | Estimate | SE | z-value | *p* | 95% CI | |
| --- | --- | --- | --- | --- | --- | --- | --- |
|  |  |  |  |  |  | lower bound | lower bound |
| *direct effect* | CES-D 🡪 frailty score | 0.06 | 0.01 | 7.21 | <0.001 | 0.04 | 0.07 |
| *indirect effect* | CES-D 🡪 left supramarginal 🡪 frailty score | 0.00 | 0.00 | 1.39 | 0.164 | -0.00 | 0.01 |
| *total effect* | CES-D 🡪 frailty score | 0.06 | 0.01 | 8.46 | <0.001 | 0.05 | 0.07 |

Table S1- Mediation model testing the role of left supramarginal gyrus thickness on the link between depression and frailty.

**S2. Mediation model testing the mediation effect of right middle frontal gyrus on the link between CES-D and Frailty score.**

The mediation model revealed no mediation of the right rostral middle frontal gyrus thickness on the link between depression and frailty (see table S2).

|  | | Estimate | SE | z-value | *p* | 95% CI | |
| --- | --- | --- | --- | --- | --- | --- | --- |
|  |  |  |  |  |  | lower bound | lower bound |
| *direct effect* | CES-D 🡪 frailty score | 0.06 | 0.01 | 7.10 | <0.001 | 0.04 | 0.07 |
| *indirect effect* | CES-D 🡪 Right rostral middle frontal 🡪 frailty score | 0.00 | 0.00 | 1.31 | 0.189 | -0.00 | 0.00 |
| *total effect* | CES-D 🡪 frailty score | 0.06 | 0.01 | 8.46 | <0.001 | 0.05 | 0.07 |

Table S2- Mediation model testing the role of right rostral middle frontal gyrus thickness on the link between depression and frailty.
